# Cross-Talk of Phosphorylation and Prolyl Isomerization of the C-terminal Domain of RNA Polymerase II

**DOI:** 10.3390/molecules19021481

**Published:** 2014-01-27

**Authors:** S. D. Yogesha, Joshua E. Mayfield, Yan Zhang

**Affiliations:** 1 Department of Molecular Biosciences, University of Texas at Austin, Austin, TX 78712, USA; E-Mails: yogeshasd@austin.utexas.edu (S.D.Y.); jmayfield@utexas.edu (J.E.M.); 2 Institute for Cellular and Molecular Biology, University of Texas at Austin, Austin, TX 78712, USA

**Keywords:** CTD, RNA polymerase II, transcription, prolyl-isomerization, phosphorylation, combinatorial regulation

## Abstract

Post-translational modifications of the heptad repeat sequences in the C-terminal domain (CTD) of RNA polymerase II (Pol II) are well recognized for their roles in coordinating transcription with other nuclear processes that impinge upon transcription by the Pol II machinery; and this is primarily achieved through CTD interactions with the various nuclear factors. The identification of novel modifications on new regulatory sites of the CTD suggests that, instead of an independent action for all modifications on CTD, a combinatorial effect is in operation. In this review we focus on two well-characterized modifications of the CTD, namely serine phosphorylation and prolyl isomerization, and discuss the complex interplay between the enzymes modifying their respective regulatory sites. We summarize the current understanding of how the prolyl isomerization state of the CTD dictates the specificity of writers (CTD kinases), erasers (CTD phosphatases) and readers (CTD binding proteins) and how that correlates to transcription status. Subtle changes in prolyl isomerization states cannot be detected at the primary sequence level, we describe the methods that have been utilized to investigate this mode of regulation. Finally, a general model of how prolyl isomerization regulates the phosphorylation state of CTD, and therefore transcription-coupled processes, is proposed.

## 1. Introduction

The three RNA polymerases (Pol I, II, III) in eukaryotic systems have different transcriptional roles [1,2]. The workhorse Pol II transcribes all protein-coding mRNAs as well as some non-coding RNAs, whereas Pol I transcribes most of the ribosomal RNAs and the main function of Pol III is the synthesis of small RNAs such as tRNAs [1,2]. Though the three RNA polymerases share high similarity in their subunit compositions and catalytic domains, a unique C-terminal domain (CTD) comprised of multiple heptad repeat sequences [3] is only found in Pol II [3]. The CTD is important as it acts as a scaffold that coordinates the Pol II transcription process with other cellular events such as cell cycle regulation and DNA repair [4,5,6,7,8,9].

From fungi to humans, the CTD consists of tandem repeats of the heptad consensus sequence YSPTSPS with its overall length correlating roughly to complexity of the organism; CTDs range from 17 repeats in *Plasmodium* *falciparum* [10] to 52 in vertebrates [7,8,9]. Conservation of the consensus sequence also varies dramatically among species: yeast has one of the most conserved CTDs with 21 out of the 26 CTD repeats fitting the consensus motif, while the *Drosophila* CTD contains only two repeats out of 42 that faithfully replicate the conserved heptad. A minimal amount of repeats must be retained (usually around half of the normal number) by each species for cell viability, presumably by supporting transcription and its associated processes [11].

## 2. Covalent Modification of CTD

The recruitment of various regulatory factors to the CTD choreographs the progression of transcriptional initiation, pausing, elongation, mRNA co-processing and termination [12]. How does such a seemingly simple heptad repeat sequence encodes such complicated functionality? Although this question still baffles researchers, an important component in transcriptional regulation are covalent post-translational modifications of the CTD with the best studied being phosphorylations. Indeed, the CTD is highly enriched in amino acids that can be phosphorylated and *in vivo* the CTD has been found in both hypo- and hyper-phosphorylated states [13,14,15]. Five out of the seven residues in the heptad repeat can be modified by phosphorylation with the phosphorylation states of Ser2 and Ser5 playing an essential roles during Pol II transcription termination and transcription associated processes [16]. The CTD is unphosphorylated for the form of Pol II that enters into a promoter (with the assistance of Pol II general transcription factors). After the assembly of the pre-initiation complex (PIC), the phosphorylation of Ser5 residues of the CTD seems to coincide with the clearance of promoter and the start of elongation. As Pol II progresses towards the 3' end of a gene, Ser5 phosphorylation level drops while Ser2 phosphorylation picks up and becomes predominant. At Pol II completes the termination process, all CTD phosphorylations are eventually cleared by CTD phosphatases to regenerate unphosphorylated Pol II molecules that may enter the pool ready for another round of transcription [16,17].

In addition to the major sites of Ser2 and Ser5, a variety of novel modifications along the CTD have been identified in recent years. These discoveries were made possible by the development of novel CTD specific antibodies and revealed new phosphorylation sites [18,19,20] such as Tyr1 phosphorylation, which has subsequently been linked to transcription termination [21]. Likewise, phosphorylation at Thr4 was observed and found to play a role in transcription termination of non-polyadenylated mRNAs such as histones [22], and in transcription elongation [18]. Ser7 phosphorylation plays an essential role in snRNA transcription [19,20]. Additionally Arg, which sometimes replaces Ser7 in mammalian CTD sequences, can be modified by methylation [23]. These recent discoveries suggest an even more sophisticated CTD code mediated by post-translational modifications, yet how these modifications affect CTD-regulatory enzymes and transcription requires further investigation.

## 3. Non-Covalent Modification on CTD

A non-covalent post-translational modification of the CTD that plays an important role in transcription is proline isomerization. The only residues in the CTD consensus sequence that are not subject to phosphorylation are Pro3 and Pro6, immediately following the essential Ser2 and Ser5 residues, respectively. Therefore, during transcription the CTD becomes enriched with phosphorylated Ser(P)-Pro motifs that are the recognition site for an essential switch in signaling mediated by prolyl isomerase Pin1 [24,25]. For example, each molecule of the human Pol II CTD has more than 100 sites that can contain a phosphoryl-Ser-Pro motif [14,26] and are potentially subject to modification by Pin 1.

**Figure 1:**
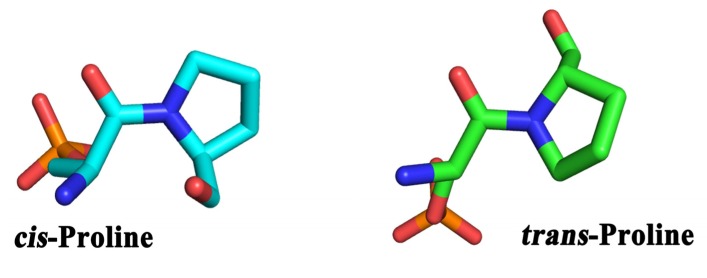
The isomerization of proline residues. *cis*- and *trans*-conformations of proline residue when the residue in front is phosphorylated serine.

The partial double bond character of peptide bonds results in two possible configuration: *cis* and *trans* (Figure 1), depending on their dihedral angle. The *trans* isomer is almost exclusively favored in 19 out of the 20 natural amino acids due to lower steric conflict. However, unique to proline, a bond between the R-group delta carbon and adjacent N-terminal nitrogen results in an organic amide and a bulky circularized side chain, which has an increased propensity for the Xaa-Prolyl bond to be in the *cis* isomer form (10%–15%). This prolyl peptide bond can spontaneously rearrange, but the kinetic energy barrier for the *cis*/*trans* isomerization is greater than 88 kJ/mol [27], making it a slow process. The isomerization process can be influenced by the phosphorylation states of flanking residues [28,29,30]. We will use the term “conformation” in this review when referring to the two isomeric structures allowed for the N-terminal peptide bond of proline, as it is relatively easy (compared to other amino acids) to convert from the *trans* form to the *cis* arrangement without the need to completely break the partial double bond.

**Figure 2:**
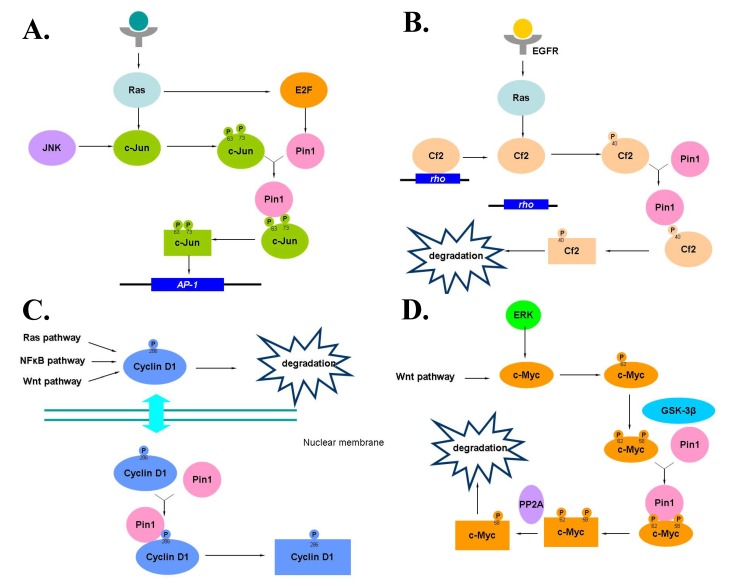
Pin1 functions as a kinetic switch in signal transduction. (**A**) c-Jun is a component of the transcription activator protein 1 (AP-1) which requires phosphorylation by Jun N-terminal kinase/stress-activated protein kinase (JNK/SAPKs) followed by the action of Pin1 to become fully active as a transcription regulator [35], (**B**) In *Drosophila*, Chorion factor 2 (CF2) is degraded as a result of epidermal growth factor receptor (EGFR) signaling which promotes the phosphorylation of CF2 at a single site. *Drosophila* Pin1 homologue, Dodo, can then act on CF2, targeting it for proteasomal degradation [34]. (**C**) Pin1 stabilizes Cyclin D1 in the nucleus, preventing its translocation to the cytosol, which would subsequently target it for degradation [35]. (**D**) c-Myc, a cell-cycle regulator, undergoes phosphorylation by ERKs. This initial phosphorylation stabilizes c-Myc. GSK3β activates and phosphorylates c-Myc at a secondary site. The initial site is then dephosphorylated by protein phosphatase 2A (PP2A), which destabilizes the protein and results in its degradation. PP2A requires the action of Pin1 to convert c-Myc into an ideal substrate [35].

Pin1 and the yeast homologue Ess1 are proline isomerases that catalyze *cis*/*trans* prolyl peptide bond inter-conversions. Interestingly, Pin1 only recognizes targets when the serine/threonine preceding the proline is phosphorylated [31]. The target specificity of Pin1 situates it as a key mediator between post-translational modification and signal transduction since key transcriptional regulators, including CTD kinases and phosphatases, recognize the same sequence motif [32]. The active sites of downstream enzymes that bind to the Pol II CTD, such as phosphatases, transcription termination factors, or capping enzymes, can be specific for only one prolyl isomerization state. Thus the isomerization activity of Pin1 can overcome the rate limiting step of prolyl *cis*/*trans* conversion and quickly replenish substrate pools. Because of this, Pin1 functions as a kinetic switch that diverts signaling pathways and, as a result, affects the enzymatic activity, cellular location, protein degradation and gene expression of cells (Figure 2), with more details reviewed in recent articles [33,34,35].

**Figure 3:**
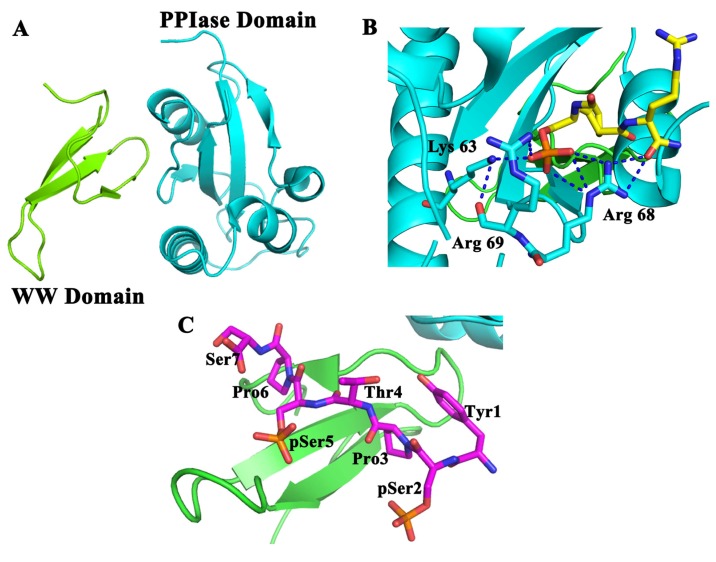
The structure of human prolyl isomerase Pin1. (**A**) The domain architecture of human prolyl isomerase Pin1 with WW domain colored green and PPIase domain colored cyan, (**B**) The active site of Pin1 PPIase domain in complex with substrate analogue (PDB ID: 3TCZ) [49], Lysine 63, Arginine 68, and Arginine 69 contribute to phosphoryl peptide substrate recognition, C) WW domain of Pin1 (green) recognizing phosphoryl-peptide substrate (PDB ID: 1F8A) [45].

Pin1 is a 163 amino acid polypeptide that can be divided into two domains based on topology: an N-terminal WW domain that provides substrate recognition and C-terminal peptidyl-prolyl *cis*/*trans* isomerase (PPIase) domain (Figure 3A) [36]. The active site of the PPIase domain is highly conserved throughout the entire parvulin family, the subfamily of proline isomerases to which Pin1 belongs [31]. The active site was identified in the structure of human Pin1 complexed with a low affinity dipeptide and is composed of a phosphate binding pocket, a prolyl binding pocket, and reaction center [36]. At the entrance of the active site, three basic residues (Lys63, Arg68 and Arg69) form a cluster that mediates binding of phosphorylated physiological targets (Figure 3B). This positively charged triad is highly conserved throughout Pin1 homologues in a variety of species and provides a selective filter in substrate recognition, explaining the three magnitude higher preference of Pin1 for phosphorylated over unphosphorylated substrates [31]. Upon peptide binding, a 70° rotation in the loop connecting α1 and β1 closes on the active site, provide a lid to isolate the prolyl peptide to undergo isomerization [37]. The residues spatially arranged around the prolyl-peptide (Cys113, His59, His157 and Ser154) are believed to facilitate the isomerization reaction. The proposed mechanism of Pin1, based on the dipeptide complex structure, includes the deprotonation of Cys113 by His59 which nucleophilically attacks the carbonyl carbon of the substrate peptide, resulting in tetrahedral intermediate formation between enzyme and substrate [36]. The spatial arrangement of the Pin1 active site has high homology with cysteine proteases and is observed to be the most efficient member of the prolyl isomerase family [38,39,40].

Pin1 activity helps regulate the transcription of mRNA precursors and Pol II-stimulated pre-mRNA processing *in vivo* [41,42,43,44], presumably through the replenishing of *cis*-prolines in the CTD. The structure of human Pin1 bound to a double phosphorylated CTD repeat gave the first glimpse of how CTD is recognized by Pin1 [45]. This structure is also the first report of the elusive CTD, which was the region not resolved in Pol II structures [46,47]. The structure showed that the binding of CTD by Pin1 was mediated through the WW domain of Pin1, a binding module at the N-terminal domain [48]. Even though both Ser2-Pro3 and Ser5-Pro6 could fit the bill as Pin1 substrates, it has been shown that only Ser5-Pro6 is recognized [45]. The biological and structural results of Pin1 on Pol II function supports a model that the WW domain acts to bind Pin1 to the CTD and confers one dimensional movements along the CTD during which the PPIase domain encounters proline sites and catalyzes their isomerization [49].

## 4. Erasers, CTD Phosphatases

Pin1 mediated prolyl isomerization plays a significant role in regulating Pol II CTD phosphatases [49]. Since Pin1 preferentially recognizes substrates with proline residues immediately following phosphorylated Ser5, its activity has the greatest impact on Ser5 phosphatases. Two well characterized examples of transcription regulation through prolyl isomerization are found in the Ser5 phosphatases, Ssu72 and Scp1 [50,51].

### 4.1. Ssu72

The eukaryotic Ssu72 (Suppressor of sua7-1 clone 2) is a phylogenetically conserved Ser5 CTD phosphatase [52,53] that was first identified in yeast genetic experiments where it was shown to impact the formation of the transcriptional PIC [54]. Further investigation revealed that Ssu72 is also involved in transcription elongation [55], mRNA processing [52] and termination [56,57]. Mutation at the active site of Ssu72 causes the accumulation of phosphorylated Ser5 on CTD. This identified the enzyme’s role in CTD Ser5 dephosphorylation [53].

**Figure 4:**
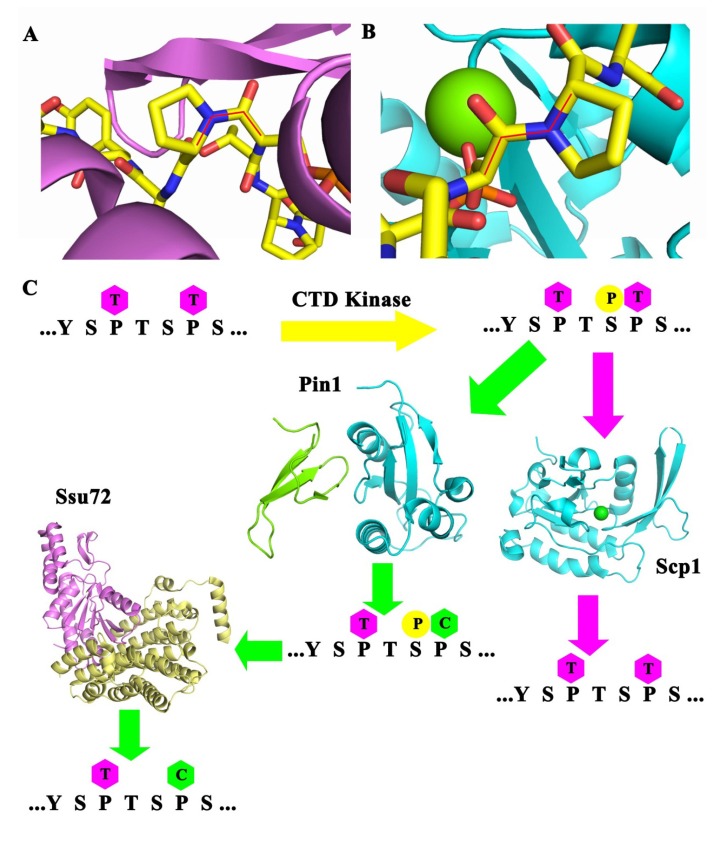
Regulation of Ser5 CTD phosphatases by prolyl isomerase. (**A**) Ssu72 in complex with a CTD phosphoryl-peptide with *cis*-proline (PDB ID: 4IMJ) [58]. (**B**) Scp1 in complex with a CTD phosphoryl-peptide with *trans*-proline (PDB ID: 2GHT) [50]. *cis*- and *trans*-conformations are highlighted in red. (**C**) Model of the regulation of CTD Ser5 phosphatases by Pin1. The activity of *cis*-specific phosphatase Ssu72 is upregulated by Pin1 isomerase activity. Yet, *trans*-specific phosphatase Scp1 bypasses Pin1 regulation.

Ssu72 utilizes a cysteine-based phosphate transfer mechanism similar to the tyrosine phosphatases [58,59]. Surprisingly, when the complex structure of Ssu72 with a Pol II CTD peptide fragment was determined [51,58,60], it was found that Pro6 of the bound peptide was in the *cis*-proline form, making Ssu72 the first *cis*-specific phosphatase reported (Figure 4A). The local geometry of the Ssu72 active site provides favorable interactions with the CTD peptide to maintain the tight β-turn for the Pro6 to adopt the *cis*-form [51]. In particular, an intra-molecular hydrogen bond between the hydroxyl group of Thr4 and the carbonyl of Pro6 further stabilized the conformation [51]. However, this intramolecular hydrogen bond is dispensable since its loss will not abolish Ssu72 recognition. This finding emphasized the importance of induced-fit model that the active site configurations of CTD enzymes prompt the CTD conformation recognized [58].

The selectivity of Ssu72 towards *cis*-proline, which is a minor species in the proline conformation pool, makes the availability of *cis*-proline a major determinant of its phosphatase activity. This requirement explains the previously identified functional interaction of Ssu72 with proline isomerase Pin1/Ess1 [44]. Since the auto-conversion of *trans*- to *cis*-proline is slow, the *cis*/*trans* isomerization becomes the rate limiting step for the reaction catalyzed by Ssu72 (Figure 4C) [25,32]. Now, proline isomerases (e.g., Pin1 and Ess1) catalyze the *cis*/*trans* conversion and thus may contribute to rebalancing the substrate pool for Ssu72 [25]. This hypothesis is greatly supported by experimental data where the addition of Pin1 to Ssu72 kinetic assays produces a three-fold rate enhancement for the apparent enzymatic activity of Ssu72 (Figure 4C) [51,61].

Recently it has been shown that Ssu72 can also dephosphorylate phosphoryl-Ser7 of the CTD consensus sequence *in vivo* [62,63]. The *in vitro* phosphatase activity of Ssu72 towards Ser7 is about 4,000 times weaker than its activity against Ser5 using synthetic peptides [64]. The crystal structure of Ssu72 recognizing the CTD peptide with only Ser7 phosphorylated revealed that the peptide is orientated 180° from the mode of recognition towards phosphoryl-Ser5 with the N- and C-termini reversed [64]. The peptide bonds connecting Pro6 to the proceeding serines in this crystal structure are in the *trans* form. The question of how prolyl isomerase activity modulates Ssu72’s action on Ser7 dephosphorylation is still not fully answered. Western blotting assays suggest that the addition of Ess1, the yeast homologue of Pin1, produces an apparent enhancement of dephosphorylation by Ssu72 on a GST-CTD doubly phosphorylated at Ser5 and Ser7 by THIIH [64]. However, the phosphoryl-specific antibodies of CTD are very sensitive to the phosphorylation state of flanking residues and, making the quantification of phosphorylation at Ser7 impossible. Better identification and quantification of phosphoryl-Ser7 using methods such as mass spectrometry will be necessary to conclude definitively about the impact of prolyl isomerase activity on Ser7 phosphorylation state. This would be particularly interesting because, unlike any substrate of Pin1 or Ess1, the proline residue precedes the phosphorylated residue rather than following it.

### 4.2. Scp1

The effect of proline isomerase activity on other CTD Ser5 phosphatases is dramatically different from *cis*-specific Ssu72. Small CTD phosphatases (Scps) are a family of three homologues that display strong activity and selectivity towards CTD phosphorylated at Ser5 [65]. Scps are localized to the nucleus of non-neuronal cells and have been shown to play a vital role in silencing the expression of neuronal genes [66]. Scps prevent neuronal differentiation of stem cells primarily through association with the REST/NRSF protein complex at RE-1 elements in undifferentiated stem cells and non-neuronal cell types [66]. Because of its activity in neuronal silencing, Scps have been proposed as a target for neural regeneration [67]. The crystal structure of Scp1 bound to a CTD peptide revealed a hydrophobic pocket interacting with Pro3 as a determinant for Ser5 selectivity (Figure 4B) and both Pro3 and Pro6 exhibit *trans* conformations. Computational modeling of *cis*-proline into Scp1 suggested that the incorporation of *cis*-proline in CTD would require major remodeling of Scp1’s active site, making *cis*-proline an unlikely substrate [50]. All the structural data strongly suggest that Scps are *trans*-specific phosphatases. Since the substrate for Scps appears to be the *trans*-form, the major CTD conformation species, the transition from *cis*-to-*trans* substrate is unlikely to be the rate-limiting step for the dephosphorylation mediated by Scp1 (Figure 4A). Consistently, the prolyl isomerase activity of Pin1 has little effect on the phosphatase activity of Scp (Figure 4C) [49]. In other words, *trans*-specific phosphatases can bypass Pin1 regulation (Figure 4C).

### 4.3. Fcp1

Fcp1, also known as TFIIF-stimulated CTD phosphatase I, is the first CTD phosphatase ever reported [68]. Fcp1 is composed of three domains: a phosphatase domain (conserved with that of Scp [69]) and a BRCA1 C-terminus (BRCT) domain [70] which are required for biological activity [71,72], and an Fcp1 specific helical domain [73]. Fcp1 dephosphorylates both Ser2 and Ser5 of the CTD *in vitro*, with a preference for Ser2 *in vivo* [74]. Fcp1 was shown to play a vital role in Pol II recycling, and transcription elongation through its phosphatase activity [71,75]. The Fcp1 structure from *Saccharomyces pombe* was determined and showed a similar reaction center as Scp where the nucleophilic Asp residue forms a covalent bond for phosphoryl transfer [73]. The prolyl-isomerization requirement of Fcp1 remains undetermined despite attempts to include CTD peptides in crystallization conditions, which thus far have yielded no observable electron density at the active site [70].

Indirect evidence has been presented to probe the effect of prolyl isomerization on Fcp1. It was reported that Pin1 accelerates the rate at which Fcp1 dephosphorylates Ser5 *in vitro* with no change being observed in Ser2 dephosphorylation *in vivo* [76]. However, additional work argued that prolyl isomerase activity inhibits Fcp1 activity [77]. The seemingly contradictory nature of proline-isomerization’s effect on Fcp1 activity is a complicated and yet unexplored question. For the purposes of this review, we present the following factors which might explain the source of controversy: (1) the preferred binding site for Pin1 is phosphorylated Ser5 as opposed to Ser2; Fcp1 is known to favor phosphorylated Ser2 with weak secondary activity against Ser5. The star activities of both proteins make interpretation of data difficult, (2) the inhibitory effect of Pin1 on Fcp1 might be caused by the binding of the WW domain of Pin1 blocking the Ser5 of CTD and effectively sequestering substrate from Fcp1 independent of its isomerase activity, and (3) Currently, it is impossible to measure the *cis-* and *trans*-isomer state *in vivo*. Therefore, *in vitro* conditions may not properly mimic the dynamics in a biologically relevant environment. Due to these complicating factors and contradictory data, the existence or identity of prolyl specificity for Fcp1 has yet to be established.

### 4.4. Rtr1

Putatively Rtr1 is the most recently identified Pol II CTD phosphatase, however the nature of its surmised catalytic activity is in debate [78,79,80]. Rtr1 is encoded by the *Saccharomyces cerevisiae* YER139C gene, which was shown to be required for growth at 37 °C in the presence of heat shock mimetic formamide, and has the closest sequence homology with the human Pol II associated factor (RPAP2) [81,82]. Rtr1 was originally suspected of playing a role in transcription because yeast strains deficient in Rtr1 were shown to be defective in inducible transcription from a GAL1 promoter [78]. Subsequent *in vivo* analysis found evidence that Rtr1 acts as a bona fide Ser5 CTD phosphatase [79]: (i) during transcription Rtr1 is observed to localize at coding regions of genes and is maximally enriched when Ser5 phosphorylation is diminished and Ser2 phosphorylation begins to plateau, (ii) deletion of Rtr1 leads to an accumulation of Ser5 phosphorylation along the CTD, and (iii) *rtr1* mutations produce decreases in Pol II-mediated transcription and defects in Pol II termination [79]. Although the case for Rtr1 acting as a CTD phosphatase is compelling, the primary sequence of Rtr1 exhibits no homology to any known phosphatase families [80] and more importantly, the first crystal structure of Rtr1 (derived from yeast strain *Kluyveromyces lactis)* revealed no apparent active site for phosphatase activity. However, since the crystal was obtained from a truncated version of Rtr1 lacking the last 57 amino acids toward the C-terminus, it is possible that the active site was partially absent in the recombinant protein. Even though the *in vitro* assay using general phosphatase substrate *para*-nitrophenylphosphate (*p*NPP) or CTD synthetic peptide shows no observable phosphatase activity [80], the phosphatase activity might have been also weakened due to the lack of binding partners in the assay that would target substrates to Rtr1 [80]. Alternatively, Rtr1 may not have an intrinsic catalytic activity but instead might act as a modulator/regulator of another *bona fide* Ser5 phosphatase.

## 5. Writers, CTD Kinases

The Pol II CTD kinases act upstream of and generate substrate for Pin1/Ess1 and are therefore unaffected by Pin1 mediated isomerization. However, since Ser2 and Ser5 are followed by proline residues and most kinases are sensitive to the proline isomerization state by strongly favoring the *trans-*conformation, it is logical that a proline isomerase could regulate kinase activity by isomerization of the non-phosphorylated CTD. Indeed, a cyclophilin-like prolyl isomerase SRcyp (also known as CASP10 or CARScyp [83] or hCyp89 [84]) has been implicated in the regulation of Pol II CTD kinases [85]. SRcyp contains an N-terminal PPIase and a C-terminal serine/arginine-rich (SR) domain, a module frequently found in splicing factors and RNA binding proteins [85]. SRcyp binds to the CTD through its SR domain *in vitro* and *in vivo*, representing a novel type of CTD interacting domain (CID) [85,86].

**Figure 5:**
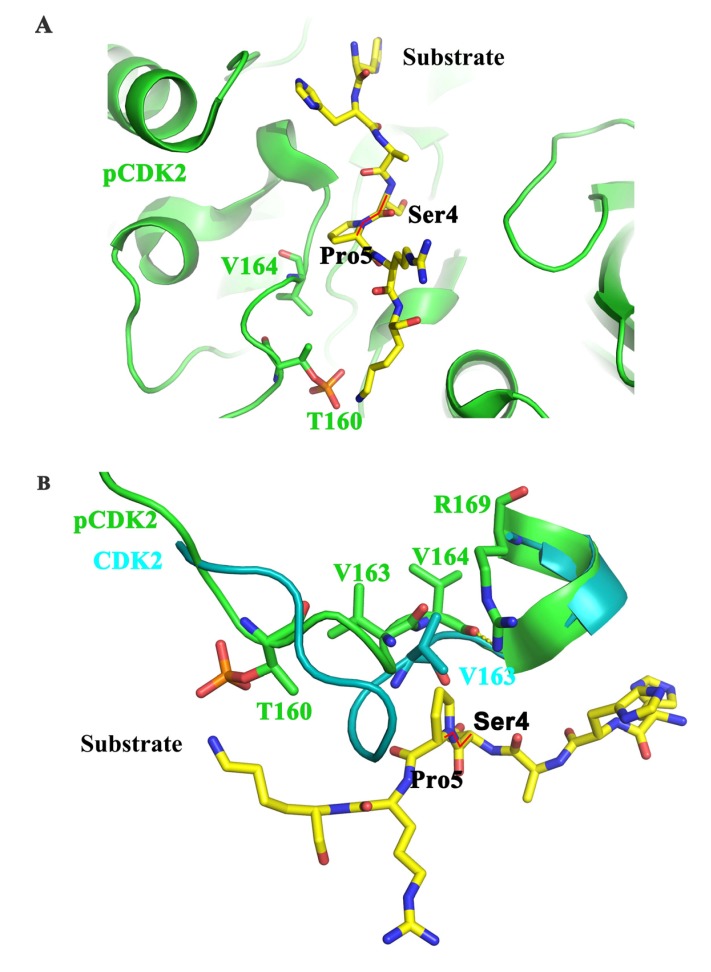
Structural specificity CDKs towards *trans*-peptide. (**A**) Phosphorylated CDK2 (green) at its activation loop on T160 shown with its substrate HHASPRK (yellow) where P+1 position is occupied by proline (PDB ID: 1QMZ) [87]. Due to an unusual left handed conformation of V164 and stabilizing effect from a hydrogen bond between V164 and R169, V164 moved away from substrate to accommodate the proline. (**B**) A magnified view of comparison of activation loops of unphosphorylated (cyan) (PDB ID: 1FIN) [90] and phosphorylated CDK2 where V163 from inactivated loop moves toward the substrate, blocking the proline at P+1. So, phosphorylated CDKs have their activation loop positioned optimally to accommodate P+1 proline in a specific *trans*-conformation.

Unlike Pin1, SRcyp regulation of the phosphorylation state of CTD hasn’t been well established. We hypothesize that the proline isomerase activity of SRcyp may facilitate *cis* to *trans* isomerization in order to promote kinase activity. So far, the well characterized CTD kinases are all cyclin-dependent kinases (CDKs), suggesting a mechanism that coordinates transcriptional events to cell cycle regulation. CDKs show strong preferences for Ser/Thr-Pro motifs, therefore, the isomerization state of the proline residue following Ser/Thr is a critical substrate specificity determinant [87,88,89]. In the crystal structure of CDK2 in complex with cyclin A and a substrate peptide, its specificity seems to be *trans*-proline only at the P+1 position (Figure 5A) [87]. In an activated CDK2, which is phosphorylated at Thr160, the Val164 residue in the activation loop establishes interactions with surrounding residues and adopts an unusual left-handed conformation in order to create a binding pocket to accommodate a *trans*-conformation proline. In non-activated CDK2, the position of Val163 pushes the activation loop towards the substrate obstructing the binding of proline in the P+1 position (Figure 5B) [90]. Comparison of both these structures strongly suggests a *trans*-specificity for CDK2 (Figure 5).

However, since CDK kinases are *trans*-specific, isomerization effects might be less dramatic in CDK kinases than *cis*-specific phosphatases. Since *trans*-proline is the dominant form in the cell, the chance of it being depleted and its availability becoming the rate-limiting step of CTD phosphorylation is low. In addition, the auto-conversion of proline isomerization in phosphorylated CTD is slower than non-phosphorylated form [32,91], therefore prolyl isomerase activity is playing a more important role in kinetic regulation when CTD is phosphorylated. Below, we will discuss a few CTD kinases with a focus on their phosphorylation specificity and proline selectivity. More detailed biological characterization can be found in a recent review [92].

### 5.1. CDK7

The phosphorylation of the Pol II CTD Ser5 is carried out at the beginning of transcription by CDK7, a subunit of the general transcription factor II H (TFIIH) [93,94,95]. CDKs require the binding of a cyclin and T-loop phosphorylation by CDK-activating kinase (CAK) for their activation [96,97]. In metazoans, CDK7 comprises a heterodimer along with Cyclin H which is then stabilized by MAT1 (ménage-à-trois 1) to form a submodule: the CDK-activating complex (CAK) of TFIIH [98,99,100,101]. As a free heterodimer, the CAK submodule is essential for activating other CDKs but upon interaction with the core TFIIH it can also phosphorylate the CTD through its CAK activity [102]. The yeast homologue of CDK7 is Kin28, which complexes with regulatory partner Ccl1 to phosphorylate the Ser5 of CTD [103,104]. Interestingly, Kin28 doesn’t possess CAK activity. The surprising absence of CAK activity in Kin28 later led to the discovery of Cak1, a CAK of yeast [105,106].

The phosphorylation of CTD mediated by CDK7 is important for the proceeding of transcription. Transcription, in general, starts with the formation of a PIC where unphosphorylated CTD interacts with the multi-protein complex Mediator as co-activator and other general transcription factors [107,108]. After the formation of PIC, the ATP-dependent activity of TFIIH unwinds the promoter at the transcriptional start site via its helicase/translocase subunits [109,110]. Phosphorylation of Ser5 by CDK7 occurs before the first phosphodiester bond [94,111,112]. Phosphorylation of Ser5 disturbs the association of Mediator complex with CTD [113,114,115] permitting promoter escape and entry into the elongation phase [12]. However, *in vitro* reconstruction of transcription can proceed in the absence of CTD or Mediator. This indicates that they are not required for the early steps of transcription [116]. In terms of its functionality in the cell, Ser5 phosphorylation seems mainly to facilitate the recruitment of pre-mRNA capping enzymes to Pol II by creating a part of the required binding epitopes on the polymerase [117,118,119,120,121].

### 5.2. CDK9

CDK9 is a component of the multi-protein complex named positive transcription elongation factor b (P-TEFb), and becomes active by binding to its regulatory subunit cyclin T [122]. Bur1/Bur2 and spCDK9/Pch1 are identified as homologues of human CDK9/cyclin T in budding and fission yeast, respectively [123,124]. A vital function of P-TFEb is to promote transcriptional elongation through its kinase activity [125,126,127]. Ser2 appears to be the preferred substrate of CDK9 as its inhibition by pharmaceutical inhibitor flavopiridol reduced Ser2 phosphorylation and impaired elongation [128]. Yet Ser2 phosphorylation by CDK9 is not necessarily the trigger of elongation. Importantly, phosphorylation of other substrates of CDK9, DRB-sensitivity-inducing factor (DSIF) and Negative Elongation Factor-E (NELF), release them from proximal pausing at the promoter to allow productive transcription elongation [125,129,130]. In budding yeast, although promoter pausing has not been observed [131], the Bur1 kinase promotes elongation through phosphorylating Spt5 and recruiting histone-modifying enzymes [132].

In addition to its Ser2 phosphorylating activity, CDK9 was also identified as Thr4 kinase [22]. CTD phosphorylation at Thr4 is newly discovered and has been implicated in transcription elongation and non-polyadenylated histone mRNA processing [18,22]. When DT40 chicken cells were treated with CDK9 inhibitors DRB and flavopirido, it was found that the levels of phosphorylated Thr4 were reduced concomitantly with Ser2 phosphorylation [22]. Currently, the question of whether or not CDK9 phosphorylates Thr4 of CTD *in vivo* is a topic of intense debate [18,22]. Since CDK9 is sensitive to inhibition by flavopiridol, it is possible that the inhibitory effect on Thr4 phosphorylation is indirectly caused by inhibition of CDK9 directed Ser2 phosphorylation. As Ser2 phosphorylation is prerequisite for Thr4 phosphorylation, blocking Ser2 phosphorylation can prevent Thr4 phosphorylation by other kinases such as PLK3 [18,133].

### 5.3. CDK12

*Saccharomyces* *cerevisiae* CTDK1 was the first Pol II CTD kinase identified [134]. CTDK1 is a three-subunit complex consisting of Ctk1 (kinase subunit), Ctk2 (cyclin subunit) and Ctk3 (a subunit with unknown function). For a long time, no Ctk1 homologues were identified in higher eukaryotes, therefore, it was widely believed that the function of both Bur1/Bur2 and Ctk1 have merged into P-TEFb during evolution as they recapitulate most of the kinase function of P-TEFb [135]. This was supported by the shared genetic interactions, as well as 43% sequence identity between CDK9 and Bur1 or Ctk1 at the kinase subunit region [126]. However, recent molecular evolution analysis showed that CDK9 is closely related to Bur1 while Ctk1 was related to human proteins CrkRS and CHED [123,136] which were later named CDK12 and CDK13, respectively [137,138]. CDK12 was characterized only recently as human homologue of yeast Ctk1 [139,140].

Ctk1 binds to Pol II and phosphorylates Ser2 [141,142]. Similarly, in higher eukaryotes, CDK12 phosphorylates the CTD *in vitro* and *in vivo* using Cyclin K as its *bona fide* cyclin partner [139,140]. Surprisingly, inhibition of CDK9 *in vivo* prevented all CTD Ser2 phosphorylation suggesting that CDK9 is the major Ser2 kinase while CDK12 only plays a limited role in CTD Ser2 phosphorylation in metazoans [143,144]. A reasonable explanation is that the inhibition of CDK9 traps Pol II in the proximal pausing stage so that downstream elongation kinases, such as CDK12, are unable to function [131,145]. Ctk1 removes the basal transcription factors from the polymerase and facilitates the transition from transcription initiation to elongation: however, such functions are not associated with its kinase activity towards Ser2 [146].

### 5.4. CDK8

CDK8/Cyclin C is the human homologue of yeast Srb10/Srb11, the yeast kinase complex that phosphorylates yeast Pol II holoenzyme *in vitro* and regulates transcription *in vivo* and has mostly been studied as a Mediator-associated kinase [147,148,149,150]. *In vitro* analysis showed that Srb10/Srb11can phosphorylate full length and synthetic CTDs at Ser5 with a similar efficiency to Kin28/Ccl1 [151]. Interestingly, CDK8 phosphorylates both Ser2 and Ser5 *in vitro* with a preference for Ser5 [152] but this has not been established *in vivo*. A kinase-deficient variant of Srb10 enhances the viability of yeast cells carrying lethal CTD truncations suggesting that Srb10 kinase negatively regulates transcription [151]. Thus it was proposed that Srb10 phosphorylates the CTD prior to the PIC, inhibiting subsequent transcription [151]. This model has gained strong support from structural studies including a stable complex of Srb8/Srb9/Srb10/Srb11 purified from yeast, namely the CDK8 kinase module (CKM) [153]. Electron microscopy studies of yeast CKM confirmed that interaction of the CKM with Mediator’s middle module obstructs the interaction of CTD-Mediator [154]. Additionally, human CKM (CDK8, cyclin C, Med12, and Med13) negatively regulate transcription by phosphorylating the CTD prior to PIC leading to disruption of Mediator-CTD interaction [151,155]. Furthermore, recombinant CDK8 was shown to inactivate CDK7/Cyclin H by phosphorylating cyclin H subunit [156], overall suggesting that CDK8 is a negative regulator of transcription.

However recent work on CDK8 suggests that it also possesses the function as a positive regulator of transcription both in yeast and humans [157]. A plethora of signaling pathways are known to utilize CDK8 as a co-activator, including p53 pathway [157], β-catenin pathway [158], the serum response network [159] and TGFβ pathway [160] implicating CDK8 in promoting transcription activity at multiple stages of transcription. The recent discovery that Mediator, which forms a complex with CDK8, can also function at elongation phase in a gene-specific fashion is a good example of its possible function in positive regulation [161]. In yeast, CDK8 is known to have a positive role in GAL4- and SIP4-mediated transcription. During GAL4-dependent transcription, CDK8 is critical for phosphorylation of Ser699 [162,163] and Sip4 phosphorylation was reduced with a mutated CDK8 [164]. The case for a positive effect on transcription mediated by CDK8 was further strengthened by the fact that recruitment of super elongation complex containing P-TEFb to hypoxia induced genes had positive effects on Pol II elongation in a CDK8-dependent manner [165].

Overall, even though both positive and negative effects on transcription have been reported for CDK8, it is still not well understood if any such effects are caused by its kinase activity towards CTD or, alternatively, other substrates during transcription. It is highly likely that both effects can be achieved in different protein contexts.

## 6. Readers, CTD Binding Proteins that Regulate Transcription

The Pol II CTD binds to more than a hundred protein factors involved in transcription regulation [166,167], linking transcription to various aspects of nuclear processes. A recent survey of the literature suggests these factors are mostly involved in transcription, mRNA processing and transport, or chromatin modification [168,169]. Only a small fraction of CTD binding proteins have their prolyl specificity defined, but of those characterized most bind to CTD with proline in the *trans* conformation [168] with the notable exception of Ssu72 [58,60] and Nrd1 [170]. The CTD proline isomerization state can determine if a protein is recruited to active transcription machinery and, therefore, plays an important role in the assembly of transcriptional complexes. In this way the prolyl specificity alters the constitution of transcriptional machinery and eventually the outcome of transcription.

### 6.1. Nrd1

Nrd1 is a RNA binding protein that plays a central role in the transcription termination of noncoding transcripts including small nuclear RNAs (snRNAs) [171], nucleolar RNAs (snoRNAs) [172], non-polyA snoRNA transcripts [173], cryptic unstable transcripts (CUTs) [174], upstream regulatory RNAs [43], and stable unannotated transcripts (SUTs) [43,175,176,177]. Nrd1 binds to the CTD in a phosphoryl-Ser5-dependent manner [174,176] and is recruited to CTD after transcription initiation and early elongation when phosphorylated Ser5 is predominant.

**Figure 6:**
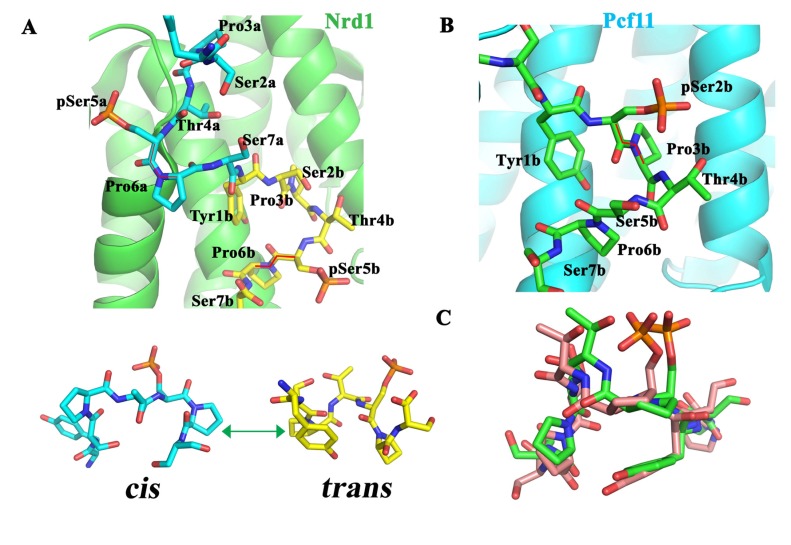
Structural comparison of Nrd1 and Pcf11. (**A**) Solution structure of Nrd1-CTD complex (PDB ID: 2LO6) showing phosphoryl-Ser5 of two consecutive CTD repeats (blue-first heptad, yellow-second heptad) bound to Nrd1 (green) [170]. The prolyl peptide between Ser5-Pro6 in first CTD repeat is in the *cis-* and second in the *trans*-conformation. The green arrow indicates an inter-conversion between the two Pro-isomers in solution. (**B**) Crystal structure of Pcf11-CTD complex (PDB ID: 1SZA) showing with Ser2 phosphorylated CTD (green) bound to Pcf11 (blue) in a *trans*-conformation [182]. (**C**) Comparison of *trans*-CTD peptides bound to Rtt103 (pink) [184] and Pcf11 (green) suggesting a conserved *trans* conformational similarity.

A recent solution structure of Nrd1 with a CTD peptide reveals that Nrd1 recognizes the CTD with Pro6 in the *cis* conformation, making it the first *cis*-specific CTD reader [170]. The Nrd1 CID-CTD structure shows the presence of two heptads, where the first one has its proline in *cis*- and the second one in *trans*-conformation (Figure 6A). This proline selectivity makes *cis*-proline a pre-requisite for the assembly of the termination complex for noncoding RNA. Indeed, this termination pathway utilizes a distinct tripartite complex of Nrd1, Nab3, and Sen1 [178] where Nrd1-Nab3-Sen1 complex is recruited to the CTD via interaction between Nrd1 and phosphoryl Ser5 [169]. Recently it was shown that *Candida* *albicans* Ess1 is necessary for efficient termination of snoRNAs [179] and it was also shown that the prolyl isomerase activity of Ess1 is required for Nrd1-dependent transcription termination of small ncRNAs [43]. Ess1 also promotes the release of Nrd1 from CTD in terminator regions [43]. These data are consistent with the notion that the conversion of *cis*- to *trans*-proline in CTD by prolyl isomerase provides a better binding site for downstream CIDs.

### 6.2. Pcf11

Protein 1 of Cleavage and polyadenylation Factor (Pcf11) is a conserved component of the yeast mRNA cleavage factor IA (CFIA), which is essential for transcription termination and 3'-RNA processing [180]. Pcf11 preferentially binds to CTD with phosphorylated Ser2, although it also binds to unphosphorylated CTD [181]. The mechanism of recognition of phosphorylated CTDs by Pcf11 was revealed by a crystal structure of Pcf11-CID in complex with a Ser2 phosphorylated CTD peptide (Figure 6B) [182]. Interestingly, the phosphate group of Ser2 was extended away from the surface without direct contact to Pcf11, partially explaining why Pcf11 can also bind to non-phosphorylated CTD (Figure 6B) [182,183]. In the crystal structure, both Pro3 and Pro6 were bound in the *trans*- conformation [182] (Figure 6B).

A solution study of the phosphoryl-CTD peptide revealed that the unbound peptide existed in a mixed *cis* and *trans* population of prolines with the *trans*-conformation as dominant form [183] but upon binding with Pcf11, only the *trans*-proline structure was observed [182,183]. This selectivity against one of the two conformers strongly underscores the importance of proline isomerization [183]. More recently, it has been proposed that Pcf11 competes with Nrd1 in binding to the CTD, and the competition is regulated by the Ess1 activity [43]; Ess1 promotes the release of Nrd1 at termination sites by converting *cis*-proline sites to *trans*-proline, which also promotes the dephosphorylation of Ser5 sites by Ssu72, and thus favors the Pcf11 binding to phosphoryl-Ser2 sites [43].

### 6.3. Capping Enzymes

The addition of a 7-methyl-Gppp cap to 5`-end of mRNA, referred to as capping, is an essential step during transcription for both the stability and translation of mRNAs [185,186]. Capping is the first modification that occurs co-transcriptionally on nascent transcripts, overlapping with CTD phosphorylation during transcription [185,186,187]. *In vivo* studies show that capping enzymes are associated with 5` end of the gene, which is facilitated by their interaction with phosphoryl-Ser5 of the CTD [119,188,189] as well as the globular body of Pol II [121]. Biochemical studies have revealed that recruitment and binding of the GTPases to CTD requires phosphorylated Ser5 [188,190]. The molecular mechanism behind this recognition was provided by crystal structures of murine, human, and fungal GTPases [191,192,193,194]. Co-crystallization of murine GTPase with an 18-amino acid CTD phosphoryl-peptide revealed that each N-terminal domain interacted with a 6-amino-acid CTD segment (phos.S_5_P_6_S_7_Y_1_phos.S_2_P_3_) where phosphoryl-Ser5 and Tyr1 provided the majority of interactions and both Pro3 and Pro6 adopted a *trans*-conformation [193]. Interestingly, in the *Candida* *albicans* capping enzyme Cgt1-CTD (4 heptads) structure, the CTD binds to the surface using two nonadjacent heptads [192]. Although mammalian and fungal GTPases utilize slightly different mechanisms for recognition of Ser5-CTD, proline residues consistently exist in the *trans*-configuration in crystal structures [193].

### 6.4. Rtt103

Rtt103 was identified as a 60 kDa protein binding to phosphorylated Ser2 CTD during an attempt to isolate novel CTD-binding proteins from yeast whole-cell extracts [195]. In addition to CTD, Rtt103 associates with Rat1, a nuclear 5' → 3' exonuclease, and its cofactor Rai1. The binding of Rtt103 CID domain to hyperphosphorylated CTD recruits Rat1 to the transcription complex [195]. Knockdown of Rtt103 or Xrn2, a human homologue of Rat1, impaired transcription termination and strongly supported the role of Rat1/Xrn2 in termination [195,196,197]. Association of Rtt103 with Rat1 restored the activity of exonuclease-deficient Rat1 emphasizing the importance of Rat1-Rtt103-Rai1 complex formation [198]. The solution structure of Rtt103 CID bound to a CTD peptide with phosphorylated Ser2 revealed that the CTD adopted a β-like conformation similar to other CTD-CID structures [182,184,199]. Comparison of CTD bound to Rtt103 with that of Pcf11 suggests that these prolyl residues also exist as the *trans*-isomer (Figure 6C) [182].

### 6.5. SCAF8

SCAF8 (SR-related CTD-associated factor 8) was initially identified through a yeast two-hybrid screen for CTD binding [86]. It was later confirmed that the C-terminal domain of SCAF8 exhibits strong binding towards doubly phosphorylated Ser2/Ser5 CTD, suggesting a function during the elongation stage [200]. Biophysical characterization showed that SCAF8 also binds to singly phosphorylated Ser2 or Ser5 and unphosphorylated CTD with weaker affinity [199]. Crystal structure of SCAF8 CID with Ser2P/Ser5P-CTD peptide revealed that CTD bound to CID in a β-turn conformation with proline residues in the *trans* form [199].

## 7. Methods to Study the Prolyl Isomeric Specificity

Since prolyl-isomerization results in conformational changes with no changes at the sequence or molecular weight levels, detection of such subtle changes can be challenging. The most direct method is the visualization of ligand conformation using X-ray crystallography. Although reliable, several aspects of this approach limit the implications regarding proline specificity. First, since X-ray crystallography reports an averaged effect of the models, the observation of *trans*-proline (the dominant species) modeled into the electron density for CTD ligand doesn’t exclude the possibility that *cis*-proline can also bind at the active site but the signal is obscured due to its small population. Second, we cannot exclude the possibility that an alternative conformation of proline can bind to the target protein but not captured in crystallization. Therefore, it is premature to conclude that a CTD protein is *trans*-proline specific simply because a *trans*-proline is modeled into the electron density for the ligand. We can only conclude that the protein is either *trans*-specific or promiscuous for both *cis-* and *trans*-proline. On the other hand, if the ligand shows a *cis*-proline conformation, it will be safer to conclude based on an X-ray structure that this protein is *cis*-specific since a minor species is captured.

The incorporation of CTDs into crystals of CTD binding proteins is usually a significant bottleneck for this approach. Since the CTD domain is an extended and disordered polypeptide, which is typically detrimental to crystal formation, researchers have to use shorter pieces of CTD repeats in the crystallographic setup (usually one to four heptad repeats). These shorter synthetic peptides usually exhibit much weaker affinity towards CTD binding proteins, making it difficult to obtain the complex structures.

NMR, which does not require crystallization, is a powerful tool to establish the binding mode of CTD peptides. NMR is highly sensitive to the local environment so differentiation of the *cis-* and *trans-*proline isomerization states is possible. Solution study of free CTD peptides revealed that both *cis-* and *trans*-conformations exist in solution and their chemical shift peaks can be differentiated [183]. When the Pcf11-CTD interaction was probed by this method, Pcf11 was clearly bound to the *trans*-conformer [183]. Compared to X-ray crystallography, NMR resolves the problem of averaging effects and allows studies in a larger time scale and in a site-specific manner [183]. The role of proline isomerization is vital in making a peptide template suitable for the specific CID to bind to the CTD through *cis*/*trans* conversion [183,201,202]. NMR also overcomes the problems of low occupancy and shorter lifetime of the *cis* form [203,204].

**Figure 7:**
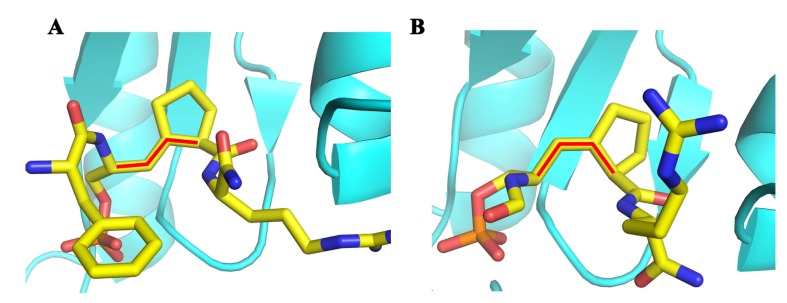
*cis*- and *trans-*locked proline analogues. (**A**) Synthetic *cis*-peptide incorporating a carbon-carbon double bond in place of the Serine 5/Proline 6 peptide bond and effectively locking the proline in the *cis*-conformation, (**B**) Crystal structure of Pin1 (cyan) complexed with a *cis*-locked phospho-peptide (yellow with *cis*-conformation highlighted in red); PDB file 3TCZ [49].

A third method has recently been developed that takes advantage of chemical probes that were generated to mimic the double bond property of prolyl peptide [49,205]. In this series of compounds, a carbon-carbon double bond replaces the prolyl peptide and locks the isomerization state of the peptide bond to mimic either the *cis* or *trans* state and completely negates the equilibrium between these forms. For example, a locked isostere has been used successfully to mimic the substrates of prolyl isomerase Pin1. By using a *cis* and *trans* locked isostere, the ground states of Pin1’s substrate recognitions have been captured (Figure 7A,B) [49]. Therefore using peptide mimetics with locked proline isosteres, it is possible to determine specificity for the proline isomerization state of CTD binding proteins. By testing the kinetic activities or binding affinities of CTD enzymes on these CTD mimetics, we can conclude if a CTD enzyme is *cis*-specific, *trans*-specific or non-discriminative with regard to proline isomerization state.

## 8. Hypothetical Model for the Effect of Prolyl Isomerization on Transcription

**Figure 8:**
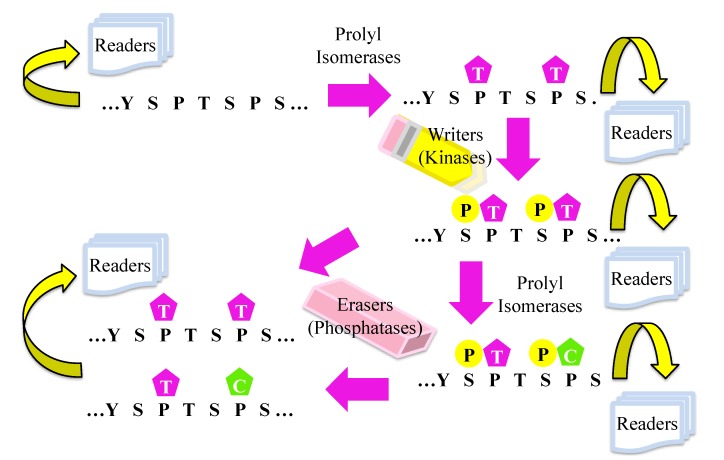
A general model of the cross-talk between phosphorylation and proline isomerization. Phosphorylation and proline isomerization can act together to dynamically regulate CTD function.

A combinatory regulation mechanism based on the variable phosphorylation and other modifications—the CTD code—emerges from our current knowledge. As discussed above, post-translational modifications on different sites of the CTD do not seem to function in any singlistic mode. Instead, modifications appear to affect each other (Figure 8). Prolyl isomerization of the heptad repeats can directly affect the phosphorylation state of the CTD by making specific Ser5-Pro6 and possibly Ser2-Pro3 registers a better or worse substrate for CTD kinases or phosphatases. Prolyl isomerases can rapidly shape the balance of the substrate pool. For instance Pin1 can help replenish the minority *cis*-proline pool, and thus regulate *cis*-specific kinases or phosphatases via substrate availability. On the other hand, *trans*-specific or non-selective kinases and phosphatases can bypass the regulation by prolyl isomerases since they are exposed to an ample substrate population (Figure 8). Combined with phosphorylation, prolyl isomerization of the CTD seems to orchestrate the recruitment of regulatory proteins to the Pol II transcription machinery and thus influences the overall outcome of transcription.
